# Motion Contrast, Phase Gradient, and Simultaneous OCT Images Assist in the Interpretation of Dark-Field Images in Eyes with Retinal Pathology

**DOI:** 10.3390/diagnostics14020184

**Published:** 2024-01-15

**Authors:** Mircea Mujat, Konstantina Sampani, Ankit H. Patel, Ronald Zambrano, Jennifer K. Sun, Gadi Wollstein, R. Daniel Ferguson, Joel S. Schuman, Nicusor Iftimia

**Affiliations:** 1 Physical Sciences, Inc., 20 New England Business Center, Andover, MA 01810, USA; patel@psicorp.com (A.H.P.); dan@fergusonrd.com (R.D.F.); iftimia@psicorp.com (N.I.); 2 Beetham Eye Institute, Joslin Diabetes Center, Boston, MA 02115, USA; konstantina.sampani@joslin.harvard.edu (K.S.); jennifer.sun@joslin.harvard.edu (J.K.S.); 3 Department of Medicine, Harvard Medical School, Boston, MA 02115, USA; 4 Department of Ophthalmology, New York University School of Medicine, New York, NY 10017, USA; ronald.zambrano@nyulangone.org (R.Z.); gadi.wollstein@nyulangone.org (G.W.); 5 Department of Ophthalmology, Harvard Medical School, Boston, MA 02115, USA; 6 Wills Eye Hospital, Philadelphia, PA 19107, USA; jschuman@willseye.org

**Keywords:** adaptive optics, retinal disease, scanning laser ophthalmoscopy, optical coherence tomography, diabetic retinopathy

## Abstract

The cellular-level visualization of retinal microstructures such as blood vessel wall components, not available with other imaging modalities, is provided with unprecedented details by dark-field imaging configurations; however, the interpretation of such images alone is sometimes difficult since multiple structural disturbances may be present in the same time. Particularly in eyes with retinal pathology, microstructures may appear in high-resolution retinal images with a wide range of sizes, sharpnesses, and brightnesses. In this paper we show that motion contrast and phase gradient imaging modalities, as well as the simultaneous acquisition of depth-resolved optical coherence tomography (OCT) images, provide additional insight to help understand the retinal neural and vascular structures seen in dark-field images and may enable improved diagnostic and treatment plans.

## 1. Introduction

Dark-field imaging methods have gained increased interest recently in high-resolution retinal imaging. First introduced more than a decade ago [1], offset aperture has essentially opened up a new type of tissue imaging which revealed retinal structures that had not been seen before in the confocal channel of adaptive-optics scanning laser ophthalmoscopes (AO-SLO). Instead of detecting backscattered photons through the confocal aperture, in the offset mode, photons that were scattered mostly forward and then reflected back by the most reflecting layers located below the target of interest are collected. Phase objects that have a minute refractive index difference with respect to their surroundings, and large particles, deflect photons mostly forward and generate very low contrast in the confocal channel: there is, however, a lot of information in these forward-scattered photons that can be detected and analyzed through offset apertures. The initial demonstration of the offset aperture method has enabled the visualization of the retinal neural structure and vasculature with exquisite details and of the blood vessel walls in particular.

Split-detection imaging [2], using two offset apertures on opposite sides of the illumination spot, was introduced shortly after offset aperture imaging. The split image is obtained by subtracting the two offset images and then dividing by their sum. This method removes common background photons and provides enhanced imaging contrast compared to the individual offset images. The first demonstration of split-detection in ophthalmology was used to visualize the inner segments of the photoreceptors. Differential phase contrast methods have been introduced in microscopy using central illumination and split-detection [3,4,5], which are essentially the same as those employed here in ophthalmology, as well as using split-source (asymmetric illumination) and central detection [6].

Several groups have demonstrated multi-offset approaches [7,8,9,10] in which four or more offset apertures are used simultaneously or sequentially to image the same retinal location. Repositioning the offset aperture sequentially at multiple locations relative to the illumination spot [9] provides increased contrast and extraordinary details of retinal structures; however, the method is very difficult to use in the clinic. The free-space optics arrangement [10] for the simultaneous acquisition of four offset apertures and the confocal is difficult to align and maintain. The fiber bundle detection [7,11] method enables robust and simple simultaneous imaging in the confocal and the four offset channels. This method has been used to reveal cellular details of the blood vessel wall in an isotropic way, free of directionality artifacts that affect individual offset or split-detection methods. Detailed analysis of the vessel wall enables the quantification of the wall-to-lumen ratio and the identification of compromised wall integrity locations which may potentially lead to the formation of microaneurysms and/or blood leaks [11]. The retinal tissue response to controlled light flicker through neurovascular coupling [12] results in dilation of retinal vessels; however, this process can be impaired by diabetes and other vascular diseases [13,14], and therefore, the precise quantification of vasodilation may provide valuable information on the health of the retina. All these capabilities enabled by high-resolution multi-offset retinal imaging and analysis have diagnostic value and may open new avenues for monitoring disease progression and treatment plans.

The commonly accepted interpretation of offset-aperture imaging is that photons scattered mostly forward by microstructures in the focal plane are back-reflected by deeper retinal layers and collected through larger apertures that are shifted laterally from the illumination location. In general, the deeper layers reflect in a diffuse manner and do not affect light collection through the offset aperture. This interpretation generally assumes that the optical path between the illumination spot and the offset aperture is rather isotropic, and therefore, the structural information shown in offset-aperture images mostly illustrates refractive index discontinuities located in the focal plane. Such an assumption is often not valid, as we show in this paper, in situations where retinal deposits or intraretinal/subretinal fluid distort the light collection geometry and appear as blurred spots in eyes with retinal pathology. The proper interpretation of such images may require additional information that can be provided by cross-sectional imaging such as OCT [15,16] and by the additional analysis of simultaneous multi-offset aperture imaging.

The main purpose of the study was to demonstrate the ability of multimodal high-resolution multi-offset retinal imaging technology to visualize retinal structures not visible with other imaging methods, particularly in eyes with retinal pathology, and to enable the definition of biomarkers for retinal diseases. The analysis presented here facilitates the proper interpretation of offset-aperture imaging. In addition, this study enabled a cellular-level analysis of blood vessel walls that provides quantification of the geometric characteristics of the vessels [11]. The wall-to-lumen ratio has diagnostic value, and additional results from this study will be presented in a future publication.

## 2. Materials and Methods

### 2.1. Imaging Systems

The main imaging system used in this study has been described previously [7,11]. A photograph of the multimodal adaptive-optics (AO) retinal imaging platform (MAORI) in a clinical setting (Figure S1) is included in the Supplementary Materials to illustrate the instrument’s footprint. MAORI was used to collect data at the Beetham Eye Institute of the Joslin Diabetes Center in Boston. Our imaging approach combines OCT with multi-offset/confocal SLO within the same optical layout. Both imaging modalities, SLO and OCT, are AO-corrected (AO-SLO, AO-OCT) [17,18,19]. The multi-channel SLO detection scheme uses five optical fibers (four offset and one confocal) to collect light simultaneously with the OCT B-scan. These fibers are mounted together within the ferrule of a fiber optic connector placed in the SLO detection plane and all five imaging channels are aligned together in a simplified manner, compared to individual channels in free-space optics arrangements. An additional advantage of the fiber bundle detection configuration is that the five detectors placed at the other ends of the individual fibers can be placed conveniently away from the main optical path, therefore simplifying hardware constraints. The four offset images can be combined in multiple ways, providing pairs of orthogonal split-detector images which then enable phase reconstruction and calculation of the phase gradient [11], which can then be used for additional tissue characterization. Motion contrast is obtained by calculating the standard deviation image (STD) after registering a stack of about 100 images and provides vasculature mapping without the use of additional contrast agents.

A compact adaptive-optics retinal imager (CAORI) was used at the Advanced Ophthalmic Imaging Laboratory at the New York University (NYU) School of Medicine in New York. Initially developed as an adaptive-optics line-scanning ophthalmoscope (AO-LSO) [20], CAORI has been converted recently to a flying-spot ophthalmoscope with the same capabilities as MAORI, including both AO-SLO and AO-OCT channels and the fiber bundle detection approach for simultaneous acquisition of the four offset apertures, the confocal image, and the OCT B-scan. A photograph of CAORI (Figure S2) is included in the Supplementary Materials to illustrate the very small instrument footprint, suitable for a clinical setting. Both MAORI and CAORI, illustrated here, have been engineered to a very compact form as compared to other research instruments of similar capabilities. Both prototypes contain the same imaging capabilities, including common path AO-SLO and AO-OCT channels, and a very similar wavefront sensor, motorized patient interface assembly for easy eye positioning in the instrument pupil, motorized fixation display, and motorized OCT delay line. Both instruments use the same SLO detection assembly, consisting of the fiber bundle, off-the-shelf detectors (Thorlabs), and multichannel digitizer (Alazar). The only notable difference between the two instruments is the form factor. As can be seen in the two Figures S1 and S2 additional engineering provided a more compact form for CAORI compared to MAORI for a better-suited clinical footprint. CAORI takes up about half of the space needed by MAORI, comparable to clinical OCT instruments.

### 2.2. Human Subjects and the Imaging Procedure

An observational study for evaluating adults with type 1 diabetes (T1D) was conducted at the Beetham Eye Institute of the Joslin Diabetes Center (JDC) in Boston under IRB approval. Exclusion criteria included non-diabetic retinal pathology, pupillary miosis, inability to dilate, prior panretinal photocoagulation, and media opacities. Before the AOSLO/OCT imaging session, each subject underwent mydriasis, and ultrawide fundus photography (UWF) during a single visit. Diabetic retinopathy (DR) severity was graded by certified graders on colored UWF based on the Early Treatment Diabetic Retinopathy Study (ETDRS) classification system. Thirty four (34) subjects, including five (5) controls and twenty nine (29) diabetic patients ranging from no DR to severe DR, were imaged at JDC. A second part of the study was conducted at the NYU School of Medicine and was approved by the NYU School of Medicine’s IRB. Forty two (42) subjects were imaged at NYU. All participants signed informed consent before being enrolled to the study.

### 2.3. Non-Confocal Imaging

**Figure 1:**
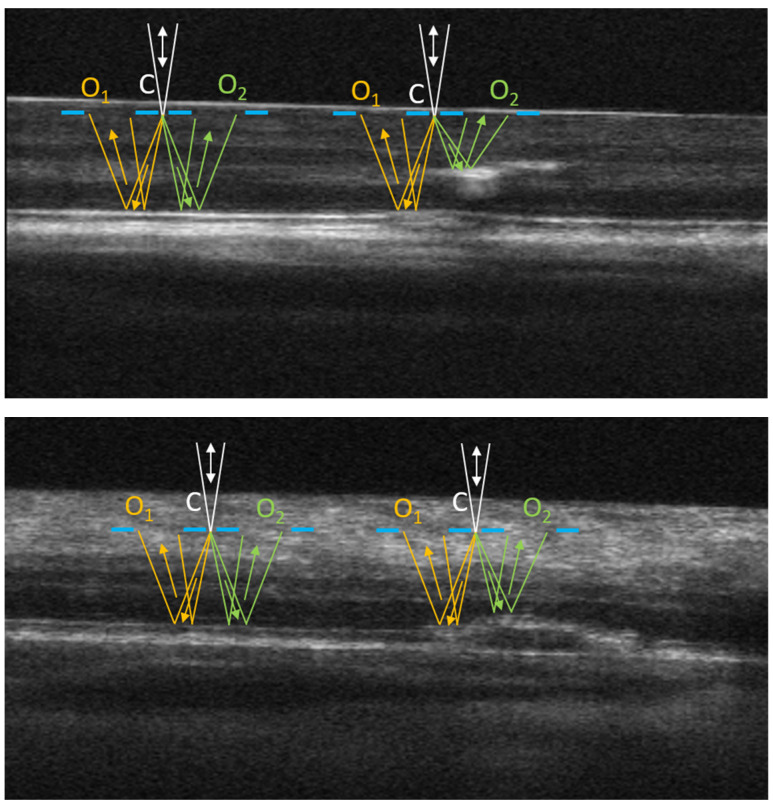
Diagram of light paths in offset-aperture imaging for different eye pathologies. O_1_, O_2_—offset apertures, C—confocal aperture. The double white arrow indicates the illumination/detection path in the confocal channel; the yellow and green arrows indicate the detection path in the offset aperture channels.

Examples of the optical path in the non-confocal, offset-aperture imaging configuration are shown in Figure 1. Two OCT B-scans illustrate the layered structure of the retina, including intraretinal/subretinal deposits in the eyes, with retinal pathology. Light focused at the virtual location of the confocal aperture (C) is scattered according to the scattering phase function associated with the refractive index inhomogeneities (microstructures) in the focal plane. Some photons are directly backscattered and collected through the confocal aperture in the confocal imaging channel. Other photons are forward-scattered and then through multiple scattering events including, reflections on deeper layers of the retina (photoreceptor complex and the retinal pigment epithelium (RPE)), are collected through the offset apertures (O_1_ and O_2_, as illustrated in Figure 1) if their trajectories fit within the numerical aperture, the collection angle of the offset apertures. If the optical path between the illumination spot and the two offset apertures is relatively isotropic, statistically, O_1_ and O_2_ collect a similar amount of photons and their ratio depends only on the anisotropies present in the illumination spot in the focal plane. This scenario is illustrated in the left-hand-side diagrams of the two images in Figure 1. However, structural disturbances along the optical path can misbalance this light collection geometry. As shown on the right-hand-side diagrams in Figure 1, subretinal and/or intraretinal abnormalities can change the photon collection through O_2_, compared to O_1_, in a way that does not reflect the microstructures located in the focal plane. The result could be either a brighter or a darker spot that can obscure the imaged structure from the focal plane in one of the offset images, more than in the other three (only two offset apertures are illustrated in Figure 1 of the four simultaneously collected). If these structural disturbances are deep, outside of the imaging beam depth of focus, these spots appear blurred in the offset images and cannot be interpreted from the SLO images alone. Simultaneous acquisition of OCT B-scans may help elucidate their location, and therefore, potentially, their origin.

The split-detector image (split 1) is calculated as the difference of two offset aperture images divided by their sum. The two offset apertures are located symmetrically on opposite sides of the illumination spot, which also coincides with the confocal aperture, as illustrated in Figure 1. A second split-detector image (split 2) is obtained in the same way using a pair of offset apertures located along a direction perpendicular to split 1 (in a plane perpendicular to the image plane shown in Figure 1). Split-detector images have been interpreted as phase derivatives in oblique back-illumination [21] and differential phase contrast [22] microscopy arrangements, and, therefore, the magnitude of the phase gradient (MPG) can be calculated as the square root of the sum of squared split-detector images [11]. Motion contrast is provided by the flow of erythrocytes through the retinal vasculature. We generally acquire about 100 images at the same retinal location, and the automatically selected least distorted images are registered and aligned [23]. The mean of the aligned stack provides an improved definition of retinal microstructures, while the average standard deviation of the split images (STD) provides vasculature mapping through motion contrast. Blood flow generates large intensity fluctuations and therefore large standard deviation, while stationary tissue exhibits low standard deviation.

## 3. Results

**Figure 2:**
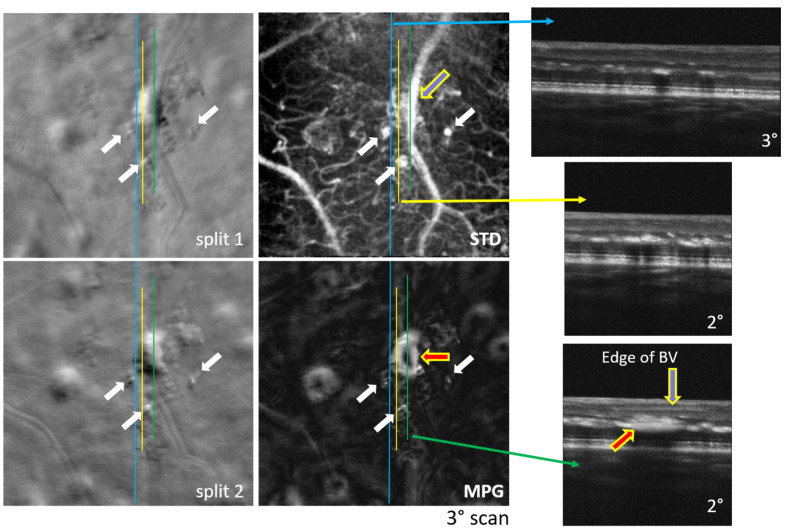
Split-detection (split 1 and 2), motion contrast (STD), phase gradient magnitude (MPG) images, and OCT B-scans at retinal locations, indicated by the blue, yellow, and green lines. The red arrow indicates the location of intra-retinal deposits; the blue arrow outlined in yellow indicates the location of the blood vessel along the OCT B-scan; the white arrows indicate micro-aneurysms.

**Figure 3:**
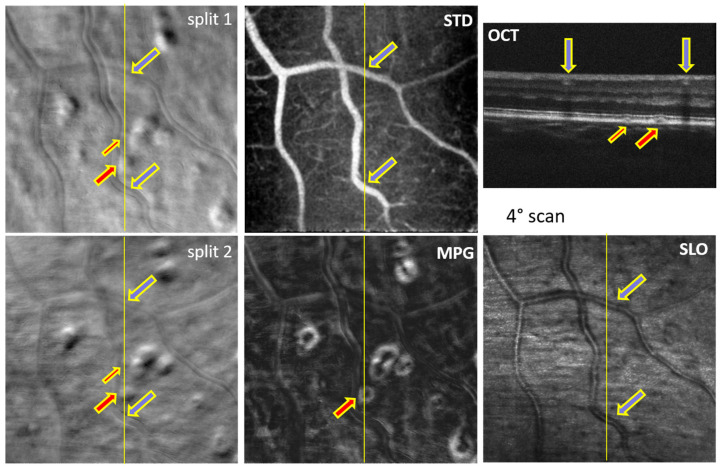
Split-detection (split 1 and 2), motion contrast (STD), phase gradient magnitude (MPG), confocal (SLO) images, and OCT B-scan at the retinal location indicated by the yellow line. The red arrows indicate the location of small drusen; the blue arrows indicate the locations of the blood vessels intersecting the OCT B-scan.

**Figure 4:**
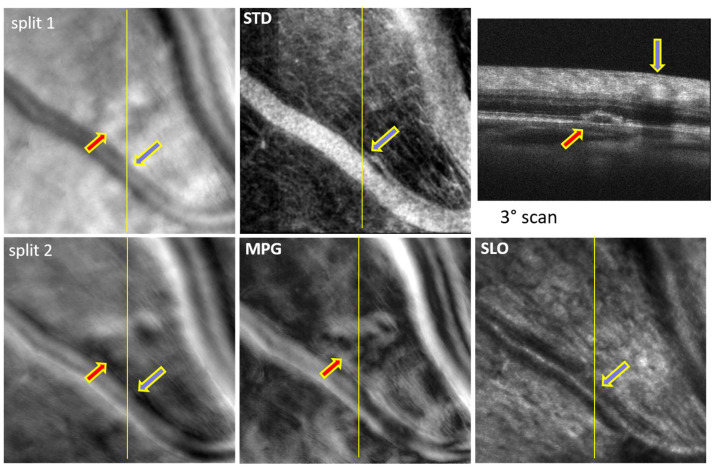
Split-detection (split 1 and 2), motion contrast (STD), phase gradient magnitude (MPG), confocal (SLO) images, and OCT B-scan at the retinal location indicated by the yellow line. The red arrow indicates the location of an elevation in the RPE; the blue arrow indicates the location of the blood vessel intersecting the OCT B-scan.

**Figure 5:**
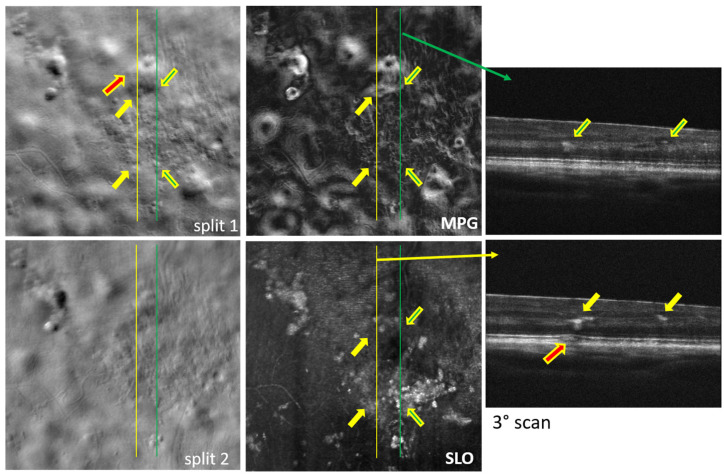
Split-detection (split 1 and 2), phase gradient magnitude (MPG), confocal (SLO) images, and OCT B-scans at the retinal location, indicated by the yellow and green lines. The red arrow indicates the location of a small drusen; the yellow and green arrows highlighted in yellow indicate the locations of the intra-retinal deposits visible in the OCT B-scans.

**Figure 6:**
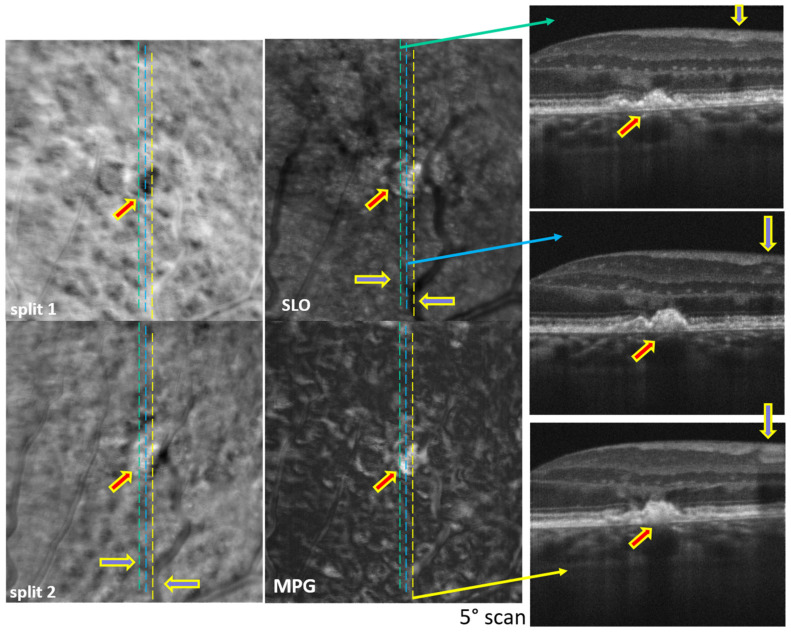
Split-detection (split 1 and 2), phase gradient magnitude (MPG), confocal (SLO) images, and OCT B-scan at the retinal locations, indicated by the green, blue, and yellow dotted lines. The red arrow indicates the location of a large drusen; the blue arrows highlighted in yellow indicate the location of the blood vessel intersecting the OCT B-scans.

**Figure 7:**
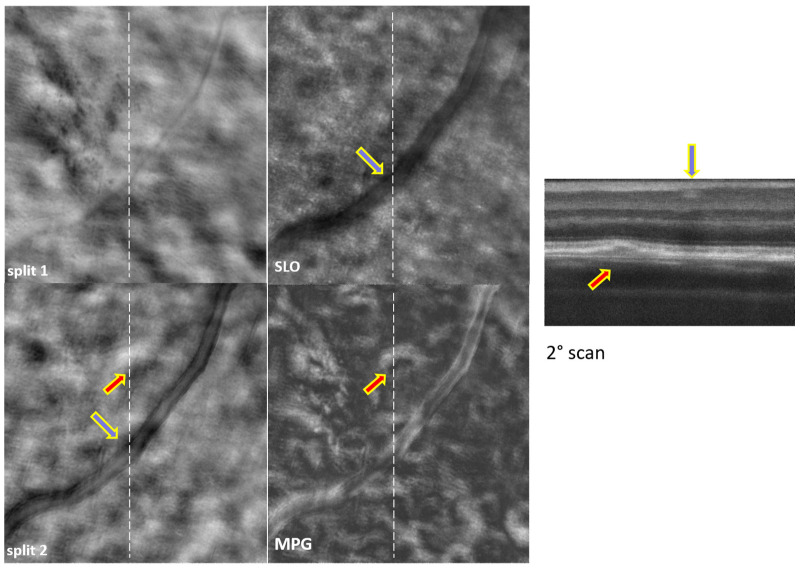
Split-detection (split 1 and 2), phase gradient magnitude (MPG), confocal (SLO) images, and OCT B-scan at the retinal location indicated by the white dotted line. The red arrow indicates the location of a drusen; the blue arrow indicates the location of the blood vessels intersecting the OCT B-scan.

Examples of simultaneously acquired OCT and SLO images are shown in Figure 2, Figure 3, Figure 4, Figure 5, Figure 6 and Figure 7, illustrating the complementarity of simultaneous OCT/SLO imaging. RPE disruptions, retinal deposits, or intraretinal fluid appear in offset/split SLO images as blurred spots that frequently cannot be interpreted from the SLO images alone. When the focal plane is located in the upper retina, although the capillary/vessel wall details appear sharp, the deeper retinal structures and lesions appear blurred and may overlap with the image structures from the focal plane. The OCT image helps in localizing and interpreting these structures.

The images in Figure 2, Figure 3, Figure 4 and Figure 5 were obtained using MAORI at the JDC from the left eye (Figure 2 and Figure 3) and the right eye (Figure 4 and Figure 5) of a 31-year-old, white female with 18 years of type 1 diabetes and mild nonproliferative diabetic retinopathy (NPDR).

Figure 2 shows a large number of exudates deposited primarily in the outer plexiform layer (OPL), as can be seen in the OCT B-scans. A large deposit, indicated by the red arrow, seems to also extend into the inner nuclear layer (INL). Segments of blood vessels that are oriented perpendicular to the split direction appear sharp in the split images, indicating that the focal plane is located in the RNFL, where the larger vessels are. Therefore, the exudates located in the OPL appear blurred in the split images, as they are located deeper than the focal plane. One blood vessel in the STD image runs partially parallel to the OCT B-scan, indicated by the green line, and the bottom edge of the vessel can be seen in the bottom right OCT image in Figure 2, below the blue arrow. The white arrows indicate micro-aneurysms (MAs). The flow of blood through the MAs generates motion contrast and they appear bright in the STD image. Similar structures in the split and MPG images can be differentiated from the MAs since they have no correspondence in the STD image; therefore, they are stationary deposits, hard exudates, or fluid pockets.

Figure 3 shows two small drusen, indicated by red arrows. They are not visible in the SLO images since they are deep below the focal plane and are very small. They have no blood flow and, therefore, do not produce motion contrast in the STD image. However, even small structural disturbances such as these two drusen can disrupt the photon collection geometry in offset imaging and appear as small blurred spots in the offset/split images as well as in the MPG image. The blue arrows indicate the location of the blood vessels that intersect the OCT B-scan and they are shown mainly as landmarks, confirming the location of the OCT B-scan within the SLO image.

Figure 4 shows a large drusen, indicated by a red arrow. The blue arrow shows the location of a large blood vessel in the RNFL, far above the drusen. The sub-retinal structural disturbance appears as a blurred spot in the split and MPG images.

Figure 5 shows a large number of exudates deposited in the INL/OPL region in addition to a small drusen/subretinal fluid spot, indicated by a red arrow. Some of the INL/OPL deposits are indicated by yellow/green arrows as they also appear in the OCT B-scans. The right green arrow in the top OCT image points to two darker spots that seem to contain clear non-scattering fluid. Their refractive index step with respect to the surroundings creates enough index inhomogeneity to produce contrast and become visible in the split and MPG images. The INL/OPL deposits appear sharper in the SLO images as they are closer to the focal plane anterior in the retina compared to the drusen/subretinal fluid spot, which is deeper and therefore appears blurred.

The images shown in Figure 6 and Figure 7 were obtained with CAORI at the NYU School of Medicine in New York from the right eye of a 68-year-old male with nonexudative age-related macular degeneration (AMD). Figure 6 contains a very large drusen in the center, indicated by the red arrow. Such a large structural disturbance deeper in the retina appears visible even in the SLO image, although it is blurred in the split and MPG images. The blue arrows indicate the location of the blood vessels in the OCT B-scan and in the SLO/split images. Similarly, Figure 7 shows a small drusen in the top-center, indicated by the red arrow. The very sharp details of the blood vessel wall, particularly in the split 2 image, confirm that the focal plane is higher up in the retina, and therefore, the drusen appears blurred.

## 4. Discussion

In both MAORI and CAORI, the horizontal line in the SLO images is generated with a resonant scanner, as in typical AO-SLO systems. The OCT A-line is located in the middle of this line (or very close to it). Both the SLO line and the OCT A-line are scanned vertically with only one galvanometer, and, therefore, the OCT B-scan is located in the center of the SLO images, as indicated in Figure 2, Figure 3, Figure 4, Figure 5, Figure 6 and Figure 7 by various continuous or dotted lines. Multiple SLO/OCT scans (consisting of ~100 frames each) are generally acquired at each retinal location. Small intentional or un-intentional fixation shifts among different scans provide OCT B-scans located close to each other, as shown in Figure 2, Figure 5 and Figure 6. In these figures, the SLO/split/STD/MPG images are very similar across different scans and we only show one of these maps at each retinal location.

OCT imaging, provided simultaneously with multi-offset SLO in MAORI and CAORI, enables the depth-resolved localization of inner layer structural distortions and facilitates an understanding the formations seen in SLO images. The focal plane for both SLO and OCT imaging can be adjusted axially as desired; however, in these investigations we are mostly interested in the blood vessels and the operator intentionally pulls the SLO focal plane anteriorly in the retina while monitoring the image sharpness of the imaged vasculature. Sharp details of the blood vessel wall and of multiple capillaries confirm that the SLO focal plane is located in the inner retinal layers; therefore, intraretinal cysts or exudates, and subretinal fluid located deeper in the retina with respect to the focal plane, can appear blurred in the split image, as a nonspecific finding, difficult to distinguish from other types of retinal pathology or to explain from the SLO image alone.

In high-resolution retinal imaging, the eye is dilated to about an 8 mm pupil diameter and the imaging beam has a 7–8 mm diameter by design. Assuming an optimal AO aberration correction, the ocular geometry allows for a best diffraction-limited illumination spot on the retina of the order of ~2–3 µm depending on the eye length, while also providing a depth of focus of ~15–20 µm, as defined by the Rayleigh range in Gaussian optics. The total retinal depth from the top of the RNFL to the RPE layer varies with the retinal location over a range of 200–500 µm, and, therefore, the sharpness of the SLO image corresponds to a very narrow slice of tissue contained within the depth of focus, while other structures located outside of the depth of focus appear blurred. The focal volume contained within the depth of focus axially and with a diameter set by the diffraction limit (the waist of the Gaussian beam) has the largest light power density within the beam path, and is expected to provide the dominant brightness in the SLO/offset images. However, given the layered structure of the retina, tissue components outside of the focal volume may be more reflective/scattering than the ones located within the focal volume, and may appear brighter, although blurred, which explains the phenomenology addressed in this paper.

All the split images shown in Figure 2, Figure 3, Figure 4, Figure 5, Figure 6 and Figure 7 illustrate a wide range of retinal microstructures of various sizes and sharpness levels. Some of them can easily be identified as capillaries or MAs in conjunction with the STD images that provide motion contrast. Some other microstructures that have no blood flow associated with them but appear bright and sharp may be located in the upper layers of the retina close to the focal plane and may represent exudates, cysts, or fluid pockets. As confirmed in the OCT images, deeper structures such as drusen or subretinal fluid appear blurred in the offset/split/MPG images, since they are far from the focal plane and affect the photon collection path expected in offset imaging configurations, as illustrated in Figure 1.

Our focus in these particular investigations was mostly on the vasculature located in the upper layers of the retina and the presence of other structural disruptions in eyes with pathology that may affect the visualization of the targeted vasculature details. However, this multimodal approach may inform medical personnel and allow them to adjust the location of the focal plane axially such that the blurred structures come to focus. Therefore, additional diagnostic information may become available beyond the initial target of this investigation and may help improve retinal health outcomes.

## 5. Conclusions

In general, in DR and other retinal vascular diseases, the focal plane of interest in dark-field imaging is located anteriorly in the retina to reveal the fine details of the retinal vasculature and of the vessel walls. In some eyes with a pathology, intra-retinal deposits, drusen, exudates, cysts, subretinal fluid, and other structural disturbances are also present and they can overlap with or even obscure the primary structures of interest. Their interpretation from SLO/offset images alone is rather difficult. Particularly in eyes with multiple structural distortions from retinal pathology, the interpretation of SLO images can be assisted by simultaneously acquired OCT images that provide additional depth-resolved contrast and by motion contrast and phase gradient images. The combined AO-SLO/AO-OCT imaging approach may provide valuable information that enables an improved diagnosis of retinal diseases and, potentially, better vision health for eyes with multiple pathologies.

## Data Availability

Data can be made available upon request subject to PSI approval. The data are not available due to privacy restriction.

## Supplementary Materials

The following supporting information can be downloaded at https://www.mdpi.com/article/10.3390/diagnostics14020184/s1, Figure S1: photograph of MAORI; Figure S2: photograph of CAORI.
