# Supplementary figures and images for: Motion Contrast, Phase Gradient, and Simultaneous OCT Images Assist in the Interpretation of Dark-Field Images in Eyes with Retinal Pathology
Source: Diagnostics (Basel). 2024 Jan 15;14(2):184. doi: 10.3390/diagnostics14020184 (PMC10814023; doi:10.3390/diagnostics14020184)

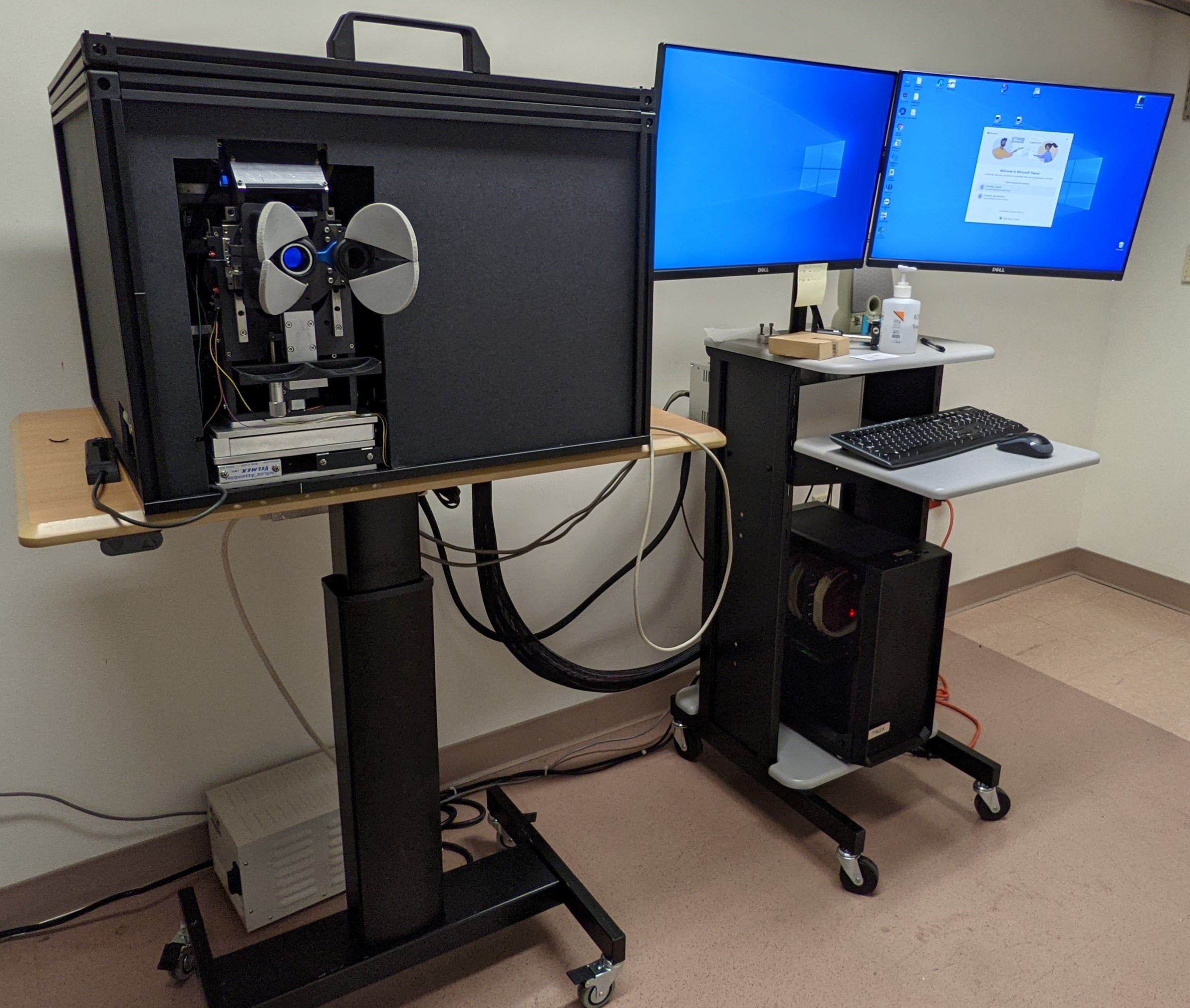

Supplement: Supplementary file 1 [file diagnostics-14-00184-s001.zip › S1.jpg]

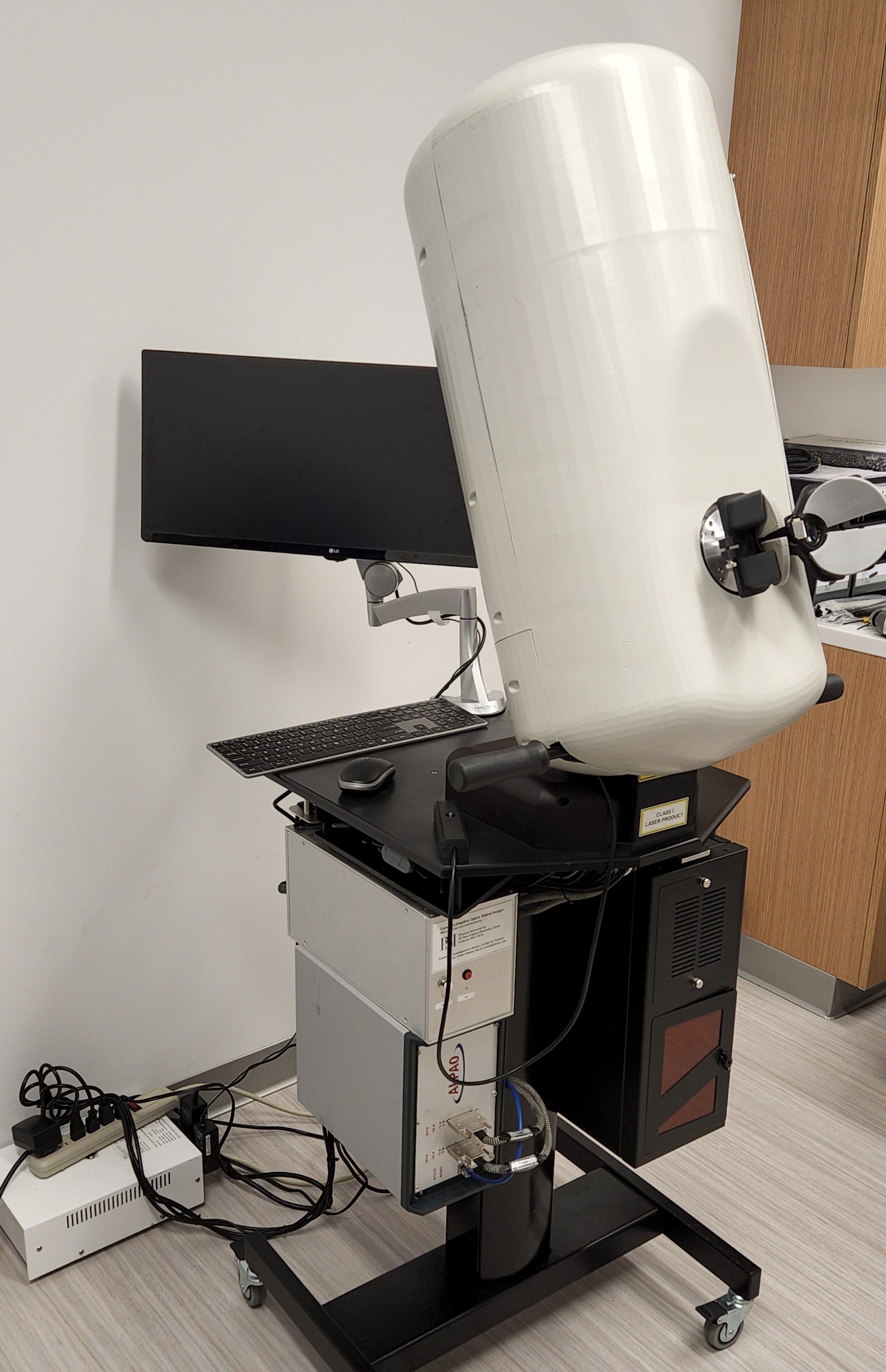

Supplement: Supplementary file 1 [file diagnostics-14-00184-s001.zip › S2.jpg]
